# Case of Empyema of the Left Plural Cavity Treated by Injection of Iodine

**Published:** 1857-11

**Authors:** Daniel Brainard

**Affiliations:** Professor of Surgery in Rush Medical College, etc


THE NORTH-WESTERN

MEDICAL AND SURGICAL JOURNAL.

(NEW SERIES.)

VOL. VI. NOVEMBER, 1857. NO. 11.

### ORIGINAL COMMUNICATIONS.

ARTICLE I.—*Case of Empyema of the left plural cavity treated by injection of iodine.* By DANIEL BRAINARD, M.D., Professor of Surgery in Rush Medical College, etc; reported by Edwin Powell, M. D.

Thomas S. Bryan, aged 16, a native of Norway, first came uuder the care of Prof. Brainard, April 15, 1857, with empyema of the left plural cavity, the result of a wound.

The previous history of the case, as given by his friends, is as follows: Some five months since the patient received a stab in the left side, between the fifth and sixth ribs, just below the nipple, with a sharp-pointed knife, which was followed by difficulty of breathing, spitting up of blood, etc. The external wound was closed with sutures and adhesive plaster, the patient was bled, purged and confined to a low diet. At the end of three weeks the wound had healed, and the patient was able to be about, but the difficulty of breathing had not entirely subsided, and he suffered more or less pain in that side.

He remained in this state for the space of two months, when a pretty copious discharge of matter took place from the wound, which had opened by ulceration. Since which time there has been a constant discharge of extremely fetid pus from the wound, and the patient was gradually becoming weaker and emaciated. The parents of the patient being in destitute circumstances, nothing was done for him during this time.

Present Condition.—The patient is now excessively thin; the skin presents a shrivelled and yellow appearance. There is a distinct bulging of the affected side, and one teacupful of fetid pus is discharged each day from the wound. The shoulder of the affected side is two inches lower than the other. He walks with the utmost caution, as the least jar or misstep gives him great pain; in fact, he is scarcely able to walk from one room to another without assistance. He is also affected with loss of appetite, chills, night sweats and diarrhœa.

Treatment.—The opening was sufficiently enlarged to admit a gum elastic tube of four inches in length, of the size of a No. 9 catheter, through which the pleural sac was washed out morning and evening, with the following solution:

| | | |
|---|---|---|
| ℞ | Iodinium, | j gr. |
| | Potassi Iodidum, | iij grs. |
| | Aqua Destillata, | ℥i |
| | M. Ft. Sol. | |

The solution was thrown in with a strong syringe, and allowed to flow out again after remaining in for a few moments. If any smarting followed the operation, the cavity was washed out with cold water. The first effect of the iodine was to change the character of the discharge to nearly healthy pus, and afterwards to lessen the amount of suppuration. No bad symptoms at any time followed the use of the injection. Iodine could be detected in the urine. The patient was, in the meantime, placed upon as generous a diet as he could take, with the use of stimulants.

As he had a very intelligent friend to take charge of him, he was allowed to go into the country to his own home, and continue this course of treatment there, after having remained under observation for two weeks.

*July 10th.*—The patient returned to the office this morning from the country. It was surprising to see the improvement that had taken place; he has gained in strength and flesh quite rapidly. Suppuration is still going on, but not much, and the discharge is of a healthy character. The injections had been faithfully used while in the country. He was advised to pursue the same plan, and again returned to his home.

*October* 10*th*.—The patient again presented himself at the office, from the country. The suppuration had entirely ceased, and the external wound had healed soundly. Instead of the mere skeleton he was seven months ago, he now presents the appearance of a sound, healthy boy.

I made a physical examination of his chest this morning and could detect no difference in the two sides.

I am not aware that injections of iodine have ever been used or recommended in this country in cases of empyema, heretofore, but such beneficial results followed their use in this case that I think we should be justified in resorting to them in similar cases.

